# Factors affecting the work ability of nursing personnel with post-COVID infection

**DOI:** 10.1038/s41598-024-60437-4

**Published:** 2024-04-27

**Authors:** Warunee Tangsathajaroenporn, Jinjuta Panumasvivat, Kampanat Wangsan, Supang Muangkaew, Wuttipat Kiratipaisarl

**Affiliations:** 1 Maharaj Nakorn Chiang Mai Hospital, Chiang Mai City, Chiang Mai 50200 Thailand; 2 Department of Community Medicine, Faculty of Medicine, Chiang Mai University, 110 Intawaroros Road, Sri Phum Subdistrict, Chiang Mai City, Chiang Mai 50200 Thailand; 3 Environmental and Occupational Medicine Excellence Center (EnOMEC), Faculty of Medicine, Chiang Mai University, Chiang Mai City, Chiang Mai 50200 Thailand

**Keywords:** Nursing personnel, Health workers, Post-COVID infection, Long COVID, Work ability, Infectious diseases, Risk factors, Occupational health

## Abstract

Post-COVID infection have raised concerns regarding their impact on nursing personnel’s work ability. This study aimed to assess the relationship between post-COVID infection and work ability among nursing personnel. A retrospective observational study from December 2022 to January 2023 involved 609 nursing personnel with a history of COVID-19 infection at a tertiary hospital. An online questionnaire measured post-COVID infection, personal and working factors, and the Work Ability Index (WAI). Long COVID was defined as the continuation or development of new symptoms 1 month post COVID-19 infection. Of 609 personnel, 586 showed post-COVID symptoms (fatigue, cough, difficulty breathing, etc.), with 73.72% in the short COVID group and 26.28% in the long COVID group. A significant association was found between WAI and post-COVID infection (aOR: 3.64, 95% CI 1.59–8.30), with the short COVID group had a significantly higher WAI than the long COVID group (mean difference 2.25, 95% CI 1.44–3.05). The factors related to work ability in the long COVID group were chronic diseases, work limitation, low job control (*P* < 0.05). Post-COVID infection, especially long COVID, adversely affect nursing personnel's work ability. Enhancing job control and addressing work limitations are crucial for supporting their return to work.

## Introduction

The COVID-19 pandemic significantly increased infections among healthcare professionals globally, with 152,888 reported cases and 1413 deaths reported between November 2019 and 2020^1^. Hospitalization or respiratory support was required in 14% and 5% of cases, respectively^2^. Recent reports have indicated the persistence of abnormal symptoms, referred to as “Post-COVID infection”, in some individuals even after two months of recovery and these persistent symptoms can affect multiple body systems^3^. Post-COVID infection can be classified into two groups: Short COVID, where abnormal symptom resolve within 4 weeks of the acute phase, and Long COVID characterized by persistent or newly emerging symptoms beyond 4 weeks post-COVID infection^4^. A Thai survey highlighted the top 10 post-COVID symptoms, including fatigue, shortness of breath, cough, insomnia, headache, hair loss, dizziness, anxiety, stress, and memory loss^5^. Several studies revealed that individuals with post-COVID infection experienced psychological impacts such as fatigue, cognitive impairment, insomnia, depression, and impulsivity^6–8^.

Approximately 10–35% of COVID-19-infected individuals experience persistent abnormal symptoms, impacting their daily lives and work^9^. A study showed that 31% of healthcare professionals experienced post-COVID symptoms, with 45% still having symptoms after three to four months. These symptoms ranged from moderate to severe, including fatigue, partial breathlessness, insomnia, and psychological abnormalities such as depression and impulsive (44%)^3^. This has led to increased stress and anxiety among healthcare professionals^10^ as well as staff shortages in healthcare settings, with some individuals returning to work while still experiencing illness^3^ and may experience reduced work capacity and presenteeism^11,12^.

Work ability, defined as the physical and mental fitness to perform tasks presently and shortly, is a holistic concept aiming for a balance between individual capabilities and job demands^13^. Work ability is crucial for the successful return to work of healthcare professionals^14^. As in a study in Italy^15^, work ability was found to be correlated with work-health balance and had a statistically significant positive correlation with job performance. Previous studies have shown a significant positive correlation between overall health, physical and mental health, and work ability among healthcare professionals^16^. The study conducted on nurses found that nurses with poor health conditions had work ability levels 14.27 times lower than those with a good health conditions^16^. In individuals affected by COVID-19, maintaining good physical activity levels showed a statistically significant positive correlation with work ability, job performance, and work productivity^17,18^. The pilot study conducted in a tertiary hospital in Chiang Mai, Thailand, revealed that 66.94% of healthcare professionals experienced the impact of post-COVID symptoms^19^. However, there is currently insufficient evidence to determine the long-term effects of COVID-19, especially for healthcare workers^3,9,20–22^.

The research aims to study the relationship between work ability and post-COVID infection in nursing professionals, comparing work abilities among Long COVID and Short COVID groups. It also assesses factors influencing work ability in nursing personnel with Post-COVID infection at the regional hospital level. The main research question was defined as whether there is a relationship between work ability and post-COVID infection among nursing personnel.

## Methods

### Study design and population

**Figure 1:**
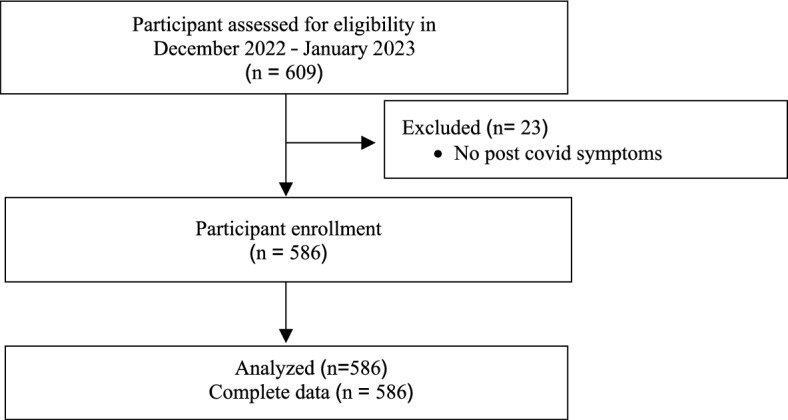
Flow diagram.

This retrospective cross-sectional observation study was conducted in a tertiary hospital in Chiang Mai, involving healthcare professionals, including nurses, practical nurses, nurse aides, and general service personnel. The study ran from 15 December 2022 - 1 January 2023, with inclusion criteria for individuals who had a history of COVID-19 infection 4 weeks before the survey, were 18 years or older, understood Thai, and willingly participated. The exclusion criteria were no post-COVID symptoms. A total of 1649 healthcare professionals who tested positive for the infection were identified by using real-time reverse transcription polymerase chain reaction (RT-PCR) or protein or antigen tests for SARS-CoV-2 (Antigen test kit, ATK, Rapid antigen test), and 609 individuals completed the online questionnaire, resulting in a response rate of 39.9%. After excluding 23 participants without post-COVID symptoms from the study, the total analyzed data included 586 participants. Study flow was showed in Fig. 1

### Data collection

The tools in this study were self-administered online questionnaires divided into four parts as follows.

1. Personal information included age, gender, marital status, having child age under 18 years old, education, weight, height, smoking, exercise, chronic diseases, individuals requiring care at home, family responsibilities, relationships with family members, living conditions, and income sufficiency.
2. Working conditions included work experience, shift work, overtime work, job characteristics, job position, skills and expertise, values and attitudes towards work, and psychosocial factors in the workplace. To evaluate psychosocial factors, the questionnaire from Phakthongsuk and Apakupakul^23^ which was modified from the Job Content Questionnaire (JCQ) was used. The JCQ content included physical demands, psychological demands, job control, social support, and workplace hazards. The scores are interpreted as low, moderate, and high based on the mean and standard deviation (± 1SD) of the sample group.
3. The Post-COVID infection questionnaire utilized a survey on COVID-19 infection and post-infection symptoms developed by the Department of Medical Services^5^. The "long COVID" group included individuals who experienced at least one abnormal symptom during the COVID-19 infection period, newly developed symptoms that persisted for at least 4 weeks, or symptoms that occurred more than 4 weeks after the infection. The "short COVID" group included individuals who experienced at least one abnormal symptom during the COVID-19 infection period that disappeared within 4 weeks^4^. Symptoms during COVID-19 infection were categorized into present viral symptoms, respiratory, sensory, and gastrointestinal symptoms. Long-COVID symptoms were categorized into cardiovascular and thoracic, general, respiratory s, neurological, dermatological, and psychological symptoms.
4. Work ability assessment was conducted using the Work Ability Index (WAI), which was translated into Thai by Kaewboonchu and Prakardkaew^24,25^. It consisted of 10 items and 7 dimensions. (1) The current work ability compared to the highest level of work ability ever achieved (2) the relationship between work ability and job demands (3) the number of current diseases diagnosed, and (4–7) estimates of the reduction in work ability due to COVID-19 illness. The total score, ranging from 7 to 49, is categorized into four groups: poor (7–27), moderate (28–36), good (37–43), and excellent (44–49). To analyze by regression analysis, work ability was classified into two groups: good work ability (scores in the good and excellent range) and poor work ability (scores in the moderate and poor range). The content validity of the questionnaire was examined by four qualified experts, yielding a content validity index (CVI) of 0.99. The questionnaire was tested with 14 nursing staff members for reliability using Cronbach’s alpha coefficient, resulting in a coefficient of 0.82.

### Ethics considerations

Study was approved by the Research Ethics Committee of the Faculty of Medicine, Chiang Mai University (Approved No. FAC MED 2565 09240). Informed consent was obtained for all participants. All methods were performed in accordance with relevant guidelines and regulations.

### Consent to participate

Informed consent was obtained from all individual participants included in the study.

### Statistic analysis and data analysis

Analysis of the relationship between work ability, post-COVID infection, and factors related to work ability was conducted using regression analysis. The work ability of nursing personnel in the long COVID group and the short COVID group were compared using a t-test for continuous variables with a normal distribution and a chi-square test for variables on nominal and ordinal scales. The statistical analysis was performed using SPSS version 24, setting significance at *P* < 0.05.

### Declaration of generative AI and AI-assisted technologies in the writing process

During the preparation of this work the authors used ChatGPT in order to improve the language. After using this tool/service, the authors reviewed and edited the content as needed and take full responsibility for the content of the publication.

## Results

### Participants' characteristics and working conditions

Out of the 609 individuals in the sample group, 586 had post-COVID symptoms. Among them, the average age was 39.3 years (SD = 12.22). The majority were female, unmarried, had one child age under 18 years old, and had a high to very high level of family responsibilities. Further details are presented in Table 1.

**Table 1:** Baseline characteristics of participants and characteristics between short COVID and long COVID groups.

Characteristics	Total (*N* = 586)	Short COVID (*n* = 432)	Long COVID (*n* = 154)	P-value
	n (%)	n (%)	n (%)	
Age (years) (mean ± S.D.)	39.3 ± 12.2	38.4 ± 12.2	41.7 ± 11.9	*0.004*^c^
Gender				*0.011*^a^
Female	519 (88.6)	374 (86.6)	145 (94.2)	
Male	67 (11.4)	58 (13.4)	9 (5.8)	
Marital status				*0.012*^a^
Single	308 (52.6)	238 (55.1)	70 (45.5)	
Married	237 (40.4)	171 (39.6)	66 (42.9)	
Widowed/divorced/separated	41 (7.0)	23 (5.3)	18 (11.6)	
Having a child aged < 18 years old				0.055^a^
Yes	247 (42.2)	172 (39.8)	75 (48.7)	
No	339 (57.8)	260 (60.2)	79 (51.3)	
Number of child < 18 years old (n = 168)				0.463^b^
1 person	105 (62.5)	75 (60.5)	30 (68.2)	
2 persons	61 (36.3)	47 (37.9)	14 (31.8)	
3 or more persons	2 (1.2)	2 (1.6)	0	
Education level				0.390^a^
Master’s degree or higher	52 (8.9)	41 (9.5)	11 (7.1)	
Bachelor’s degree	324 (55.3)	232 (53.7)	92 (59.7)	
Practical nurse/nurse aide certificate	210 (35.8)	159 (36.8)	51 (33.2)	
BMI (kg/m^2^) (mean ± S.D.)	23.5 ± 4.5	23.3 ± 4.4	24.1 ± 4.6	0.051^c^
Smoking habits				0.853^b^
Non-smoker	565 (96.4)	415 (96.1)	150 (97.4)	
Ex-smoker	13 (2.2)	10 (2.3)	3 (1.9)	
Active smoker	8 (1.4)	7 (1.6)	1 (0.7)	
Exercise				0.077^a^
None	140 (23.9)	100 (23.1)	40 (26.0)	
Non-regular (< 3 time/wks.)	365 (62.3)	264 (61.1)	101 (65.6)	
Regular (≥ 3 times/wks.)	81 (13.8)	68 (15.8)	13 (8.4)	
Chronic diseases				*0.003*^a^
No	417 (71.2)	322 (74.5)	95 (61.7)	
Yes	169 (28.8)	110 (25.5)	59 (38.3)	
Individuals requiring care at home				*0.002*^a^
No	424 (72.4)	327 (75.7)	97 (63.0)	
Yes	162 (27.6)	105 (24.3)	57 (37.0)	
Family responsibilities				0.127^a^
Very low	22 (3.8)	20 (4.6)	2 (1.3)	
Slightly low	54 (9.2)	37 (8.6)	17 (11.0)	
Moderate	194 (33.1)	150 (34.7)	44 (28.6)	
High	155 (26.5)	107 (24.8)	48 (31.2)	
Very high	161 (27.4)	118 (27.3)	43 (27.9)	
Relationships with family members				0.423^a^
Good	347 (59.2)	260 (60.2)	87 (56.5)	
Poor	239 (40.8)	172 (39.8)	67 (43.5)	
Living conditions				0.250^a^
Living alone	138 (23.5)	106 (24.5)	32 (20.8)	
Living with family	377 (64.3)	279 (64.6)	98 (63.6)	
Living with friends	71 (12.2)	47 (10.9)	24 (15.6)	
Income sufficiency				0.690^a^
Sufficient with saving	213 (36.3)	160 (37.0)	53 (34.4)	
Sufficient without saving	224 (38.2)	166 (38.4)	58 (37.7)	
Insufficient	149 (25.5)	106 (24.6)	43 (27.9)	

^a^Pearson Chi-Square.

^b^Fisher’s exact test.

^c^Unpair t-test.

BMI = body mass index.

Significant values are in italics.

In terms of work-related factors, all participants had a median (P25th–P75th) work experience of 15 (4–26) years. More than half worked in rotational shifts and worked overtime. Job control, job satisfaction, psychosocial demands, physical demands, and social support at work were mostly at a moderate level. Additional details are provided in Table 2.

**Table 2:** Working conditions between short COVID and long COVID groups.

Working conditions	Total (N = 586)	Short COVID (n = 432)	Long COVID (n = 154)	P-value
	n (%)	n (%)	n (%)	
Work experience (year), median (P25th–P75th)	15 (4–26)	13 (4–25.75)	20 (6.75–28)	*0.004*^c^
Shift work				0.102^a^
No	208 (35.5)	145 (33.6)	63 (40.9)	
Yes	378 (64.5)	287 (66.4)	91 (59.1)	
Shift works/mth., median (median (P25th–P75th) (n = 374)	13.50 (8–18)	13 (7–18)	15 (8.5–19)	0.638^c^
Overtime work				0.268^a^
No	218 (37.2)	155 (35.9)	63 (40.9)	
Yes	368 (62.8)	277 (64.1)	91 (59.1)	
Overtime (hrs./wk.), median (P25th–P75th) (n = 368)	8 (6–16)	8 (6–16)	8 (6–16)	0.754^c^
Job characteristics				*0.004*^a^
Mental	77 (13.1)	67 (15.5)	10 (6.5)	
Physical/mental	502 (85.7)	358 (82.9)	144 (93.5)	
Physical	7 (1.2)	7 (1.6)	0 (0.0)	
Skills and expertise				0.358^a^
Sufficient for the job	420 (71.7)	311 (72.0)	109 (70.8)	
Insufficient/need training	11 (1.9)	10 (2.3)	1 (0.6)	
Job requires less skill	155 (26.4)	111(25.7)	44 (28.6)	
Post-COVID-19 job modification				*0.00*^*a*^
No	492 (84.0)	377 (87.3)	115 (74.7)	
Yes	94 (16.0)	55 (12.7)	39 (25.3)	
Post-COVID-19 work limitation				*0.000*^a^
Suitable	451 (77.0)	354 (81.9)	97 (63.0)	
Limitation	135 (23.0)	78 (18.1)	57 (37.0)	
Job satisfaction				0.278^a^
High	133 (22.7)	105 (24.3)	28 (18.2)	
Moderate	329 (56.1)	239 (55.3)	90 (58.4)	
Low	124 (21.2)	88 (20.4)	36 (23.4)	
Job control				0.624^a^
High	93 (15.9)	69 (16.0)	24 (15.6)	
Moderate	450 (76.8)	334 (77.3)	116 (75.3)	
Low	43 (7.3)	29 (6.7)	14 (9.1)	
Psychological demands				0.299^a^
Low	112 (19.1)	88 (20.4)	24 (15.6)	
Moderate	418 (71.3)	306 (70.8)	112 (72.7)	
High	56 (9.6)	38 (8.8)	18 (11.7)	
Physical demands				0.564^a^
Low	113 (19.3)	87 (20.1)	26 (16.9)	
Moderate	371 (63.3)	273 (63.2)	98 (63.6)	
High	102 (17.4)	72 (16.7)	30 (19.5)	
Social support				0.060^a^
High	64 (10.9)	53 (12.3)	11 (7.1))	
Moderate	441 (75.3)	326 (75.4)	115 (74.7)	
Low	81 (13.8)	53 (12.3)	28 (18.2)	
Workplace hazards				0.264^a^
Low	98 (16.7)	70 (16.2)	28 (18.2)	
Moderate	419 (71.5)	316 (73.2)	103 (66.9)	
High	69 (11.8)	46 (10.6)	23 (14.9)	

^a^Pearson Chi-Square.

^b^Fisher’s exact test.

^c^Unpaired t- test.

Significant values are in italics.

### Post-COVID infection: long-COVID and short-COVID groups

Most of the sample group had mild symptoms (94.4%) and all of participant had respiratory symptom, including cough, sore throat, and fever. Among them, 73.7% were classified as the short COVID group, with the top three symptoms being fatigue (16.6%), cough (14.4%), and difficulty breathing (11.3%). The remaining 154 individuals (26.3%) belonged to the long COVID group, with the top three symptoms being fatigue (16.8%), cough (14.1%), and hair loss (12.2%).

The related factors that significant statistical differences between the long COVID and short COVID groups included age, gender, marital status, presence of chronic diseases, and having a home care patient (*P* < 0.05) (Table 1). Regarding working conditions, work type, and working experience were statistically significant differences between the long COVID and short COVID groups (*P* < 0.01). Additionally, the post-illness job assessment revealed that the long COVID group had a higher proportion of work limitations and a higher rate of job modifications, compared to the short COVID group (*P* < 0.01) (Table 2).

### Work ability

**Figure 2:**
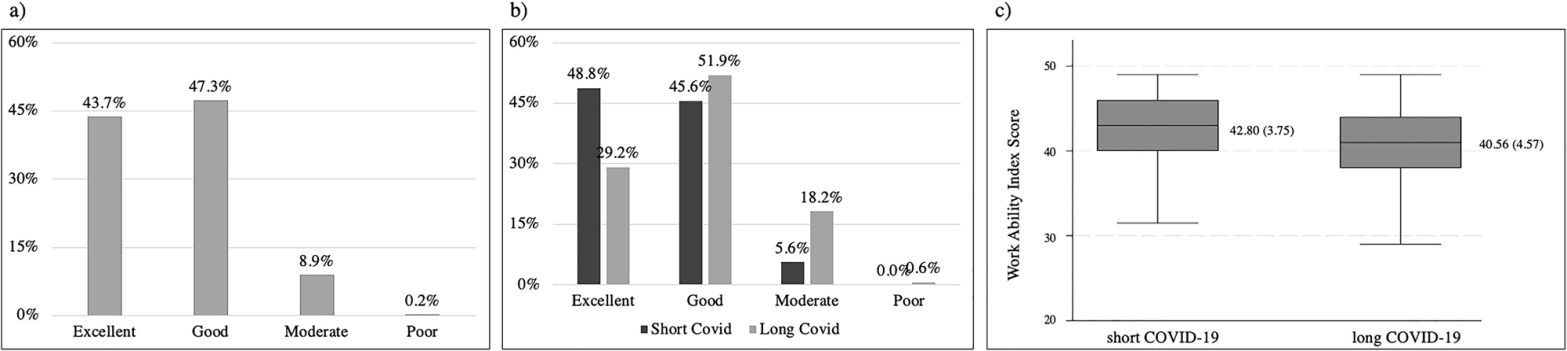
Work abilities and post-COVD-19 infection. (**a**) All post-COVID-19 participants, (**b**) Work ability between short and long COVID-19 participants, and (**c**) work ability index score between short and long COVID-19.

Participant work ability was mostly categorized as good, comprising 47.3%. In the long COVID group, the majority had a good level of work ability (51.9%), while in the short COVID group, the proportions of individuals with an excellent and good work ability were 48.8% and 45.6%, respectively. Notably, poor work ability were found only in the long COVID group, accounting for 0.6% (Fig. 2).

The average score of work ability in the Short COVID group was significantly higher than that in the Long COVID group (mean difference 2.25, 95% CI 1.44–3.05, P < 0.1).

### Factors related with work ability

The analysis using univariate logistic regression analysis revealed a significant association between post-COVID infection and work ability in the nursing personnel (OR 3.94, p < 0.01, 95% CI 2.21–7.02). When performing multiple logistic regression analysis, controlling for other factors, the long COVID group was associated with low work ability. Individuals with long COVID had a higher likelihood of having low work ability compared to the Short COVID group (aOR 3.64, p < 0.01, 95% CI 1.59–8.30). Other factors were also found to be associated with work ability including chronic diseases, poor relationships with family member, post COVID-19 work limitation, low job satisfaction, low job control, and high psychological demands (Table 3).

**Table 3:** Association between work ability and related factors in the post-COVID infection.

Variables	aOR	95% CI	*P*
Characteristics			
Gender			
Male	Ref		
Female	1.16	0.31–4.34	0.822
Age (years)	1.02	0.97–1.07	0.188
BMI (kg/m^2^)	1.07	0.98–1.16	0.127
Having child age under 18 years old (yes)	0.16	0.04–0.67	0.012*
Marital status			
Married	Ref		
Single	0.43	0.11–1.61	0.210
Widowed/divorced/separated	1.40	0.32–6.17	0.654
Education level			
Master's degree or higher	Ref		
Bachelor's degree	0.48	0.12–1.86	0.286
Certificate	0.27	0.56–1.26	0.095
Exercise			
Regular	Ref		
Non-regular	0.69	0.21–2.26	0.548
None	1.33	0.38–4.59	0.653
Chronic disease			
No	Ref		
Yes	2.85	1.18–6.91	0.020*
Relationships with family members			
Good	Ref		
Poor	3.25	1.45–7.32	0.004**
Working condition			
Post COVID-19 job modifications (yes)	1.88	0.79–4.46	0.143
Post COVID-19 work limitations (yes)	8.97	3.89–20.69	< 0.001**
Job satisfaction			
High	Ref		
Moderate	11.05	1.14–107.06	0.038
Low	24.35	2.34–252.82	0.007**
Job control			
High	Ref		
Moderate	3.06	0.42–22.08	0.268
Low	9.95	1.05–94.53	0.045*
Psychological demand			
Low	Ref		
Moderate	6.91	0.78–60.83	0.082
High	14.24	1.21–167.91	0.035*
Physical demand			
Low	Ref		
Moderate	3.21	0.60–17.19	0.173
High	1.58	0.24–10.47	0.633
Social support			
High	Ref		
Moderate	0.86	0.16–4.46	0.853
Low	0.47	0.07–2.98	0.420
Workplace hazards			
Low	Ref		
Moderate	0.52	0.16–1.66	0.269
High	0.64	0.16–2.59	0.528
COVID-19 symptoms			
Present viral symptoms (yes)	0.92	0.29–2.89	0.880
Sensory symptoms (yes)	0.76	0.28–2.03	0.580
Gastrointestinal symptoms (yes)	1.10	0.39–3.04	0.852
Long COVID	3.64	1.59–8.*30*	< 0.001**

Significant values are in italics.

**P < 0.01, *P 0 < 0.05, BMI = body mass index, Adjusted for gender, age, body mass index, marital status, education level, exercise, post COVID-19 job modification, physical demands, social support at the workplace, workplace hazards and various COVID symptoms.

### Factors association with work ability among long COVID group

The multiple logistic regression analysis was conducted to examine the factors associated with work ability specifically in the Long-COVID group. The study found that chronic diseases, post COVID-19 work limitations, low job control were significantly associated with work ability in the Long-COVID group. However, this study also found that having child under the age of 18 years was a protective factor that promoted work ability in long COVID-19 group (Table 4).

**Table 4:** Association between work ability and related factors in long COVID.

Variables	aOR	95% CI	P-value
Characteristics			
Gender			
Male	Ref		
Female	0.46	0.04–5.08	0.528
Age (years)	0.97	0.89–1.05	0.417
BMI (kg/m^2^)	1.02	0.85–1.23	0.838
Having child age under 18 year old (yes)	0.02	0–.55	0.020*
Marital status			
Married	Ref		
Single	0.03	0–0.76	0.033*
Widowed/divorced/separated	1.19	0.12–11.72	0.88
Education level			
Master's degree or higher	Ref		
Bachelor's degree	0.88	0.03–23.45	0.941
Certificate	0.09	0–4.81	0.240
Exercise			
Regular	Ref		
Non-regular	0.40	0.02–9.63	0.576
None	0.50	0.02–12.06	0.672
Chronic disease			
No	Ref		
Yes	6.67	1.36–32.49	0.019*
Relationships with family members			
Good	Ref		
Poor	0.87	0.18–4.19	0.863
Working conditions			
Post COVID-19 job modifications (yes)	1.15	0.23–5.72	0.861
Post COVID-19 work limitations (yes)	16.78	2.50–112.43	0.004**
Job satisfaction			
High	Ref		
Moderate	4.96	0.26–94.68	0.286
Low	19.74	0.88–440.82	0.060
Job control			
High	Ref		
Moderate	5.07	0.34–76.40	0.241
Low	49.86	1.16–2136.49	0.041*
Social support			
High	Ref		
Moderate	4.38	0.37–52.27	0.243
Low	1.02	0.06–17.29	0.991
Psychological demands			
Low	Ref		
Moderate	5.67	0.38–85.80	*0.210*
High	7.33	0.28–194.18	0.233
Physical demands			
Low	Ref		
Moderate	0.76	0.05–11.46	0.840
High	0.58	0.03–11.16	0.716
Workplace hazards			
Low	Ref		
Moderate	0.79	0.88–7.21	0.840
High	6.51	0.46–91.09	0.164
Long COVID symptoms			
General symptoms (yes)	3.66	0.55–24.45	0.180
CVT symptoms (yes)	3.25	0.46–22.97	0.236
Respiratory symptoms (yes)	0.49	0.10–2.39	0.383
Neurological symptoms (yes)	1.03	0.24–4.49	0.964
Psychological symptoms (yes)	4.63	0.76–28.23	0.096
Dermatological symptoms (yes)	0.59	0.11–3.09	0.529

**P < 0.01, *P < 0.05.

Significant values are in italics.

BMI = body mass index, CVT = cardiovascular and thoracic, Logistic regression adjusted for gender, age, body mass index, marital status, education level, exercise, poor relationships with family members, post COVID-19 job modifications, physical demands, social support at the workplace, workplace hazards, and various long COVID symptoms.

## Discussion

### Prevalence of post COVID-19: short and long COVID

The findings indicate a 96.2% prevalence of post-COVID symptoms among our study participants, aligning with a similar study in the general population of England and Italy, which reported a 87–90% prevalence of post-COVID symptom^26,27^. The symptoms observed in both the short COVID and long COVID groups were in alignment with prior research findings, which identified fatigue, chronic cough, and breathing difficulties as common post-COVID symptoms^5,28,29^. Contrasting our results with a study on long-COVID conditions among medical personnel in England, which reported a prevalence of 45%, we note a lower prevalence of such conditions within our study^3^. These variations in prevalence (ranging from 14 to 64%) across studies stem from inconsistent definitions, limited pathology understanding, risk factors, and diagnostic criteria, along with differing evaluation methods^5^. However, our study conducted during the Omicron outbreak with high infection rates^30^, allowing for a more comprehensive exploration of post-COVID infection.

### Work ability in post COVID-19

Notably, the sample group with post-COVID infection demonstrated a good work ability, while only 9.1% had poor work ability. These findings consisted with a 12-month follow-up study of post-COVID patients in Brazil, which reported that approximately 70–75% of their sample demonstrated good and very good work ability scores^31^. Similarly, a study among university personnel in Thailand during the COVID-19 outbreak reported 82.4% with good and very good work ability scores^32^. In contrast, a meta-analysis of nursing personnel during the COVID-19 outbreak in 2021 found a prevalence of 24.7% for poor work ability, a proportion similar to the pre-COVID-19 era^33^. Additionally, a study in India among general population with severe COVID-19 symptoms reported a lower work ability score of 16.47%^34^.

The results of this study indicate that most nursing personnel demonstrated a classification of 'good' working ability, possibly influenced by the relatively young age of the post-COVID subjects (average age 39.3 years) and the predominance of mild severity cases, with no hospital admissions. These findings are consistent with research in Brazil, where individuals infected with COVID-19, with an average age of 37.7 years^31^ and an English study, where individuals infected with COVID-19 also exhibited mild to moderate symptoms^26^.

However, despite the majority demonstrating good working ability, our study reveals that post-COVID symptoms persist and tend to impact work ability. Those who encountered work limitations post-COVID-19 recovery were more likely to exhibit poor work ability compared to those without such limitations. This finding aligns with research conducted in Switzerland, which demonstrated a significant decrease in work ability among subjects with experiencing persistent post-COVID infection and suboptimal physical or non-recovery state^35^.

The mean work ability scores were higher in the short COVID group compared to the long COVID group, emphasizing the impact of chronic illness on work ability^36^. A study conducted in England reported a 35% increase in symptoms of depression among individuals with COVID symptoms compared to their pre-COVID state. In contrast, the short COVID group exhibited a lower percentage of 18% with depression^37^. Individuals experiencing long COVID more than a month were 4.73 times more likely to take sick leave compared to those without long COVID^38^. The work ability is recognized as a significant contributor to overall life satisfaction and well-being^39^.

This study showed that COVID-19 illness factors, except for long COVID, did not significantly affect work ability. This finding aligns with a study conducted among the general working-age population in England, which similarly reported that illness severity during COVID-19 was not correlated with return-to-work factors, including work ability^26^. Meanwhile, A study on the general population of India, specifically among individuals who experienced severe COVID-19 symptoms and were hospitalized, found a lower work ability score of 16.47%^34^.

The study’s findings support the concept of work ability, representing a balance between a person's physical and mental resources and the demands of the job^13^. Individuals with post-COVID infection particularly subject who had chronic diseases and a negative view of workplace psychosocial factors are prone to reduced work ability. Additionally, work-related factors such as low job satisfaction, and limited job control were found to be associated with poor work ability. The post COVID-19 pandemic has highlighted healthcare personnel burnout and job satisfaction, which may also impact their work ability^40^. Research conducted during the COVID-19 outbreak has further substantiated this, showing that psychosocial workplace conditions significantly affect the work ability. These conditions encompass factors such as the imbalance between dedication and reward in work^32^, emotional work demands, influence on work, and work-family conflict^31^. Working across different wards and specialty may involve varying job tasks, leading to differences in job control and impacting work ability^41^.

Interestingly, having children under 18 years of age in this study was a protective factor. Previous research has established a link between having children and experiencing work-family conflicts^42^. However, within our study, the sample group exhibited positive family relationships, with 59.2% reporting good family connections. Moreover, the majority of the sample group had only one minor to care for. It is plausible that the presence of a supportive family environment and relationships may contribute to a better balance in work abilities. As highlighted in a qualitative study on emotional well-being^42^, the presence of family and close friends has been identified as a positive influence on individuals coping with COVID-19^43^ and these supportive networks could potentially play a role in promoting the work ability of the sample group. Furthermore, the responsibility of raising school-age children requires continuous learning to provide them with knowledge and diverse skills, enabling the individual to adapt to the evolving dynamics of the COVID-19 outbreak situation.

The insights from this research have the potential to inform strategic initiatives aimed at enhancing the work ability of personnel returning to work after experiencing COVID-19. This may involve the development of a comprehensive assessment system to evaluate the readiness of such personnel for reintegration into the workforce. Implementing a return-to-work program, emphasizing job control, and addressing work limitations are crucial steps to enhance the resilience of nursing personnel after COVID-19 infection, preventing poor work ability. Additionally, providing support and interdisciplinary interventions such as Cognitive Behavioral Therapy (CBT) could be considered to support the mental well-being of healthcare workers. Furthermore, our findings inform the strategic allocation of manpower during COVID-19 outbreaks and future epidemics, improving effective epidemic preparedness and response efforts.

### Strength and limitations

By pioneering research on the relationship between work ability and post-COVID infection, our study addresses a critical gap in existing research. While prior studies have predominantly focused on symptoms, tests, and treatments, our study investigated how these aspects impact an individual's work performance. The robust 39.9% response rate underscores the study's potential to offer valuable insights into the implication of post-COVID infection on occupational capabilities. Considering the focus on post-COVID, a widespread concern, our findings have potential implications beyond healthcare settings, offering insights applicable to various industries. Nevertheless, our study does have limitations. The use of retrospective questionnaires introduces the possibility of recall bias and subjectivity in participants' responses. The cross-sectional design did not track symptoms during illness, and the findings are based on individuals' perceptions, which may not accurately reflect reality. Lastly, while this study offers a comprehensive overview of nursing personnel, it still lacks exploration into potential factors such as specific specialty wards and burnout. Future research should include follow-up studies and provide evaluation from medical experts for symptom reliability. Examining deeper into other factors related to work ability, such as differences in specialty job tasks and burnout, would be beneficial. Additionally, exploring psychosocial factors beyond the workplace, such as those within family and community contexts, is recommended to guide COVID-19 prevention strategies.

## Conclusion

This study underscores the notable link between work ability and post-COVID infection among nursing personnel, particularly highlighting the potential impact of long COVID. These findings hold significant implications for human resource management and occupational health committee in healthcare, urging a thoughtful strategy to support healthcare professionals as they return to work post-COVID. Future research should focus on long-term effects and interventions for improved well-being, working conditions, and work capabilities in the post-COVID era.

## Data Availability

The data that support the findings of this study are available from the corresponding author upon reasonable request.

## References

[1] Bandyopadhyay, S. *et al.* Infection and mortality of healthcare workers worldwide from COVID-19: A systematic review. *BMJ Glob. Health.*10.1136/bmjgh-2020-003097 (2020). 33277297 10.1136/bmjgh-2020-003097 PMC7722361

[2] Wu, Z. & McGoogan, J. M. Characteristics of and important lessons from the coronavirus disease 2019 (COVID-19) outbreak in China: Summary of a report of 72,314 cases from the Chinese Center for Disease Control and Prevention. *JAMA* **323**, 1239–1242. 10.1001/jama.2020.2648 (2020). 32091533 10.1001/jama.2020.2648

[3] Gaber, T. A. K., Ashish, A. & Unsworth, A. Persistent post-COVID symptoms in healthcare workers. *Occup. Med. (Lond.)* **71**, 144–146. 10.1093/occmed/kqab043 (2021). 33830208 10.1093/occmed/kqab043 PMC8083525

[4] Tantipasawasin, P. & Tantipasawasin, S. The post-COVID condition (long COVID). *Chonburi Hosp. J.* **47**, 67–84 (2022).

[5] Department of Medical services. Post COVID-19 Care for Recovered Patients (Post-COVID Syndrome) or Long COVID for Medical Professionals and Healthcare Personnel. (2022). https://1o0.in/bf0492.

[6] Renaud-Charest, O. *et al.* Onset and frequency of depression in post-COVID-19 syndrome: A systematic review. *J. Psychiatr. Res.* **144**, 129–137. 10.1016/j.jpsychires.2021.09.054 (2021). 34619491 10.1016/j.jpsychires.2021.09.054 PMC8482840

[7] Hao, F. *et al.* A quantitative and qualitative study on the neuropsychiatric sequelae of acutely ill COVID-19 inpatients in isolation facilities. *Transl. Psychiatry* **10**, 355. 10.1038/s41398-020-01039-2 (2020). 33077738 10.1038/s41398-020-01039-2 PMC7570419

[8] Ceban, F. *et al.* Fatigue and cognitive impairment in Post-COVID-19 Syndrome: A systematic review and meta-analysis. *Brain Behav. Immun.* **101**, 93–135. 10.1016/j.bbi.2021.12.020 (2022). 34973396 10.1016/j.bbi.2021.12.020 PMC8715665

[9] van Kessel, S. A. M., Olde Hartman, T. C., Lucassen, P. & van Jaarsveld, C. H. M. Post-acute and long-COVID-19 symptoms in patients with mild diseases: A systematic review. *Fam. Pract.* **39**, 159–167. 10.1093/fampra/cmab076 (2022). 34268556 10.1093/fampra/cmab076 PMC8414057

[10] Godeau, D., Petit, A., Richard, I., Roquelaure, Y. & Descatha, A. Return-to-work, disabilities and occupational health in the age of COVID-19. *Scand. J. Work Environ. Health* **47**, 408–409. 10.5271/sjweh.3960 (2021). 34003294 10.5271/sjweh.3960 PMC8259700

[11] Li, Y., Zhang, J., Wang, S. & Guo, S. The effect of presenteeism on productivity loss in nurses: The mediation of health and the moderation of general self-efficacy. *Front. Psychol.* **10**, 1745. 10.3389/fpsyg.2019.01745 (2019). 31417468 10.3389/fpsyg.2019.01745 PMC6685003

[12] GonçalvesI, R. H. A. *et al.* (2022) Presenteeism and its influence on health personnel’s capacity for work. *Rev enferm WERJ. ***30**, e68234. 10.12957/reuerj.2022.68234.

[13] Ilmarinen, J. Work ability—A comprehensive concept for occupational health research and prevention. *Scand. J. Work Environ. Health* **35**, 1–5. 10.5271/sjweh.1304 (2009). 19277432 10.5271/sjweh.1304

[14] Truxillo, D., Cadiz, D. & Brady, G. M. COVID-19 and its implications for research on work ability. *Work Aging Retirement* **6**, 242–245. 10.1093/workar/waaa016 (2020). 10.1093/workar/waaa016 PMC7543630 38626273

[15] Previtali, F., Picco, E., Gragnano, A. & Miglioretti, M. The relationship between work, health and job performance for a sustainable working life: A case study on older manual employees in an italian steel factory. *Int. J. Environ. Res. Public Health.*10.3390/ijerph192114586 (2022). 36361464 10.3390/ijerph192114586 PMC9654428

[16] Tangsathajaroenporn, W., Kaewthummanukul, T. & Sripusanapan, A. Work ability among professional nurses in a university hospital and related factors. *Nursing J.* **39**, 152–167 (2012).

[17] Hunter, J. R. *et al.* Relationships between physical activity, work ability, absenteeism and presenteeism in Australian and New Zealand Adults during COVID-19. *Int. J. Environ. Res. Public Health.*10.3390/ijerph182312563 (2021). 34886290 10.3390/ijerph182312563 PMC8657020

[18] Hastari, S., Mufidah, E., Wahyudi, P. & Laksmita, D. Contribution of work ability and work motivation with performance and its impact on work productivity. *Manag. Sci. Lett.* **11**, 425–434. 10.5267/j.msl.2020.9.026 (2021).

[19] Nursing section organization Maharaj Nakorn Chiang Mai Hospital. Post covid survey in nursing personnel Maharaj Nakorn Chiang Mai Hospital. (Chiang Mai, 2022).

[20] Aiyegbusi, O. L. *et al.* Symptoms, complications and management of long COVID: A review. *J. R. Soc. Med.* **114**, 428–442. 10.1177/01410768211032850 (2021). 34265229 10.1177/01410768211032850 PMC8450986

[21] Akbarialiabad, H. *et al.* Long COVID, a comprehensive systematic scoping review. *Infection* **49**, 1163–1186. 10.1007/s15010-021-01666-x (2021). 34319569 10.1007/s15010-021-01666-x PMC8317481

[22] Tirelli, U., Taibi, R. & Chirumbolo, S. Post COVID syndrome: A new challenge for medicine. *Eur. Rev. Med. Pharmacol. Sci.* **25**, 4422–4425. 10.26355/eurrev_202106_26154 (2021). 34227079 10.26355/eurrev_202106_26154

[23] Phakthongsuk, P. & Apakupakul, N. Psychometric properties of the Thai version of the 22-item and 45-item Karasek job content questionnaire. *Int. J. Occup. Med. Environ. Health* **21**, 331–344. 10.2478/v10001-008-0036-6 (2008). 19228579 10.2478/v10001-008-0036-6

[24] Kaewboonchoo, O. & Ratanasiripong, P. Psychometric properties of the Thai version of the work ability index (Thai WAI). *J. Occup. Health* **57**, 371–377. 10.1539/joh.14-0173-OA (2015). 26084917 10.1539/joh.14-0173-OA

[25] Gould, R., Juhani, I., Järvisalo, J. & Koskinen, S. *Dimensions of Work Ability. Results of the Health 2000 Survey.*, (Finnish Centre for Pensions, The Social Insurance Institution, National Public Health Institute, Finnish Institute of Occupational Health., 2008).

[26] Lunt, J. *et al.* Experiences of workers with post-COVID-19 symptoms can signpost suitable workplace accommodations. *Int. J. Workplace Health Manag.* **15**, 359–374. 10.1108/IJWHM-03-2021-0075 (2022).

[27] Carfi, A., Bernabei, R., Landi, F. & Gemelli Against, C.-P.-A.C.S.G. Persistent symptoms in patients after acute COVID-19. *JAMA* **324**, 603–605. 10.1001/jama.2020.12603 (2020). 32644129 10.1001/jama.2020.12603 PMC7349096

[28] Ahmad, M. S. *et al.* “LONG COVID”: An insight. *Eur. Rev. Med. Pharmacol. Sci.* **25**, 5561–5577. 10.26355/eurrev_202109_26669 (2021). 34533807 10.26355/eurrev_202109_26669

[29] Wangchalabovorn, M. & Weerametachai, S. Prevalence of post COVID-19 conditions in SARS-CoV-2 infected patients at 3-month telephone follow-up. *Region Health Promot. Center.* **16**, 265–284 (2022).

[30] World health organization (WHO). COVID-19 WHO Thailand Weekly Situation Update No. 261–11 April 2023. (2023).

[31] Andrade, M. A., Castro, C. S. M., Batistao, M. V., Mininel, V. A. & Sato, T. O. Occupational profile, psychosocial aspects, and work ability of Brazilian workers during COVID-19 pandemic: IMPPAC Cohort. *Saf. Health Work* **13**, 104–111. 10.1016/j.shaw.2021.11.004 (2022). 34849267 10.1016/j.shaw.2021.11.004 PMC8612737

[32] Thanapop, C. *et al.* Work ability, work-related health, and effort-reward imbalance: A cross-sectional study among university staff during the COVID-19 pandemic in Thailand. *Social Sci.* **12**, 252. 10.3390/socsci12040252 (2023).

[33] Romero-Sanchez, J. M. *et al.* Worldwide prevalence of inadequate work ability among hospital nursing personnel: A systematic review and meta-analysis. *J. Nurs. Scholarsh.* **54**, 513–528. 10.1111/jnu.12749 (2022). 34918863 10.1111/jnu.12749

[34] Jain, A. *et al.* Long-term quality of life and work ability among severe COVID-19 survivors: A multicenter study. *Dialogues Health* **2**, 100124. 10.1016/j.dialog.2023.100124 (2023). 36968307 10.1016/j.dialog.2023.100124 PMC10010834

[35] Kerksieck, P. *et al.* Post COVID-19 condition, work ability and occupational changes in a population-based cohort. *Lancet Reg. Health Eur.* **31**, 100671. 10.1016/j.lanepe.2023.100671 (2023). 37366496 10.1016/j.lanepe.2023.100671 PMC10287546

[36] Abdelrehim, M., Mahfouz, E. M. & Latief, O. K. A. E. Assessment of perceived work ability and its determinants among healthcare providers. *Egypt. J. Occup. Med.* **45**, 47–64. 10.21608/EJOM.2021.143348 (2021).

[37] Fancourt, D., Steptoe, A. & Bu, F. Psychological consequences of long COVID: Comparing trajectories of depressive and anxiety symptoms before and after contracting SARS-CoV-2 between matched long- and short-COVID groups. *Br. J. Psychiatry* **222**, 74–81. 10.1192/bjp.2022.155 (2023). 36458509 10.1192/bjp.2022.155 PMC7614126

[38] Williamson, A. E., Tydeman, F., Miners, A., Pyper, K. & Martineau, A. R. Short-term and long-term impacts of COVID-19 on economic vulnerability: A population-based longitudinal study (COVIDENCE UK). *BMJ Open* **12**, e065083. 10.1136/bmjopen-2022-065083 (2022). 35998959 10.1136/bmjopen-2022-065083 PMC9402446

[39] McGonagle, A. K., Bardwell, T., Flinchum, J. & Kavanagh, K. Perceived work ability: A constant comparative analysis of workers’ perspectives. *Occup. Health Sci.* **6**, 207–246. 10.1007/s41542-022-00116-w (2022). 35574177 10.1007/s41542-022-00116-w PMC9086129

[40] Galanis, P. *et al.* Increased job burnout and reduced job satisfaction for nurses compared to other healthcare workers after the COVID-19 pandemic. *Nurs. Rep.* **13**, 1090–1100. 10.3390/nursrep13030095 (2023). 37606463 10.3390/nursrep13030095 PMC10443294

[41] Prochnow, A. *et al.* Work ability in nursing: Relationship with psychological demands and control over the work. *Rev. Lat. Am. Enfermagem.* **21**, 1298–1305. 10.1590/0104-1169.3072.2367 (2013). 24402343 10.1590/0104-1169.3072.2367

[42] Samper-Pardo, M. *et al.* The emotional well-being of Long COVID patients in relation to their symptoms, social support and stigmatization in social and health services: A qualitative study. *BMC Psychiatry* **23**, 68. 10.1186/s12888-022-04497-8 (2023). 36698111 10.1186/s12888-022-04497-8 PMC9875186

[43] Centers for Disease Control and Prevention [CDC]. Long COVID or Post-COVID Conditions. (2023). https://www.cdc.gov/coronavirus/2019-ncov/long-term-effects/index.html.

