# Author Correction: Factors affecting the work ability of nursing personnel with post-COVID infection

**DOI:** 10.1038/s41598-025-97329-0

**Published:** 2025-04-28

**Authors:** Warunee Tangsathajaroenporn, Jinjuta Panumasvivat, Kampanat Wangsan, Supang Muangkaew, Wuttipat Kiratipaisarl

**Affiliations:** 1 Maharaj Nakorn Chiang Mai Hospital, Chiang Mai City, Chiang Mai 50200 Thailand; 2 Department of Community Medicine, Faculty of Medicine, Chiang Mai University, 110 Intawaroros Road, Sri Phum Subdistrict, Chiang Mai City, Chiang Mai 50200 Thailand; 3 Environmental and Occupational Medicine Excellence Center (EnOMEC), Faculty of Medicine, Chiang Mai University, Chiang Mai City, Chiang Mai 50200 Thailand

Correction to: *Scientific Reports* 10.1038/s41598-024-60437-4, published online 27 April 2024

The original version of this Article contained errors.

The study period in the flow diagram in Figure 1 was incorrect.

The original Figure 1 and accompanying legend appears below.

**Fig. 1:**
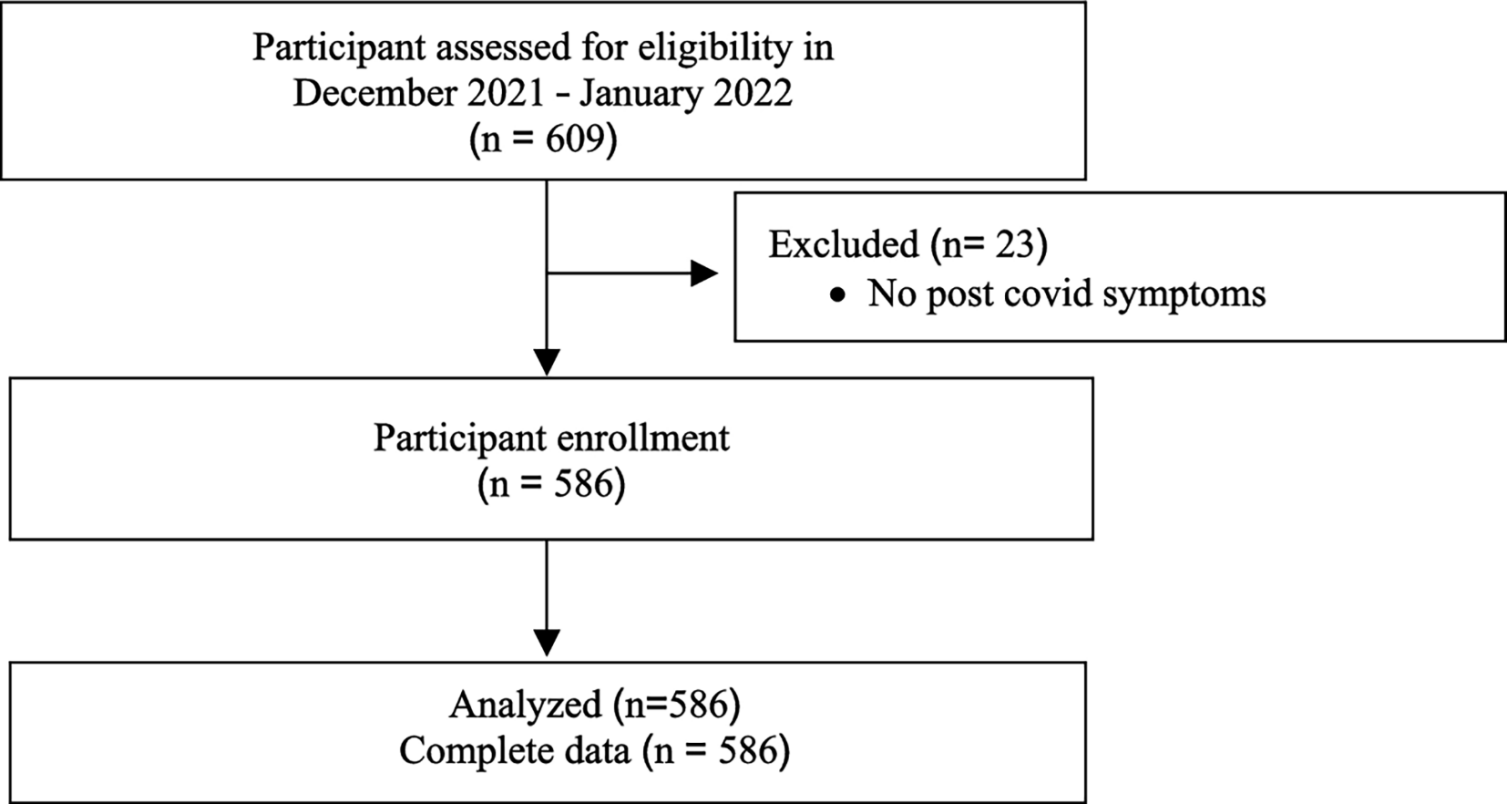
Flow diagram.

In addition, in the Methods section, under the subheading ‘Study design and population’,

“The study ran from January 1 to December 15, 2022, with inclusion criteria for individuals who had a history of COVID-19 infection 4 weeks before the survey, were 18 years or older, understood Thai, and willingly participated.”

now reads:

“The study ran from 15 December 2022 – 1 January 2023, with inclusion criteria for individuals who had a history of COVID-19 infection 4 weeks before the survey, were 18 years or older, understood Thai, and willingly participated.”

The original Article has been corrected.

